# Ministry of Health or Ministry of Labour

**Published:** 1920-11-27

**Authors:** 


### THE MATRONS' AND SISTERS' DEPARTMENT.

### Ministry of Health or Ministry of Labour.

There is general satisfaction among nurses all over the country at the resolution passed at the last meeting of the General Nursing Council in favour of removing the control of nurses' hours from the jurisdiction of the Minister of Labour to that of the Minister of Health. While strongly desiring that the conditions under which nurses are at present very generally overworked should be legally and drastically altered, nearly the whole profession has felt a recoil from being legislated for as though they were manual workers. So much was this the case that a strong body of opinion was in favour of worrying along without legislation, in the teeth of the admitted abuses, rather than surrender the professional status which has been won after years of patient endeavour. Dr. Bedford Pierce is to be congratulated on the resolution requesting the Minister of Health to prepare a Bill for regulating the Hours of Employment for nurses as the alternative to including nurses in the Bill now before Parliament.

It may be argued that the Minister of Health already has it in his power to regulate the hours which nurses may work, and, no doubt, as regards certain public institutions this is true. But members of the nursing services in a very large majority work for private employers or charitable institutions, and cannot be relieved from oppressive conditions save by legislation. Their case calls for very special treatment. Far more depends on the hours they are required to work, and the nice adjustment of rules applicable to their varied lives, than the relief of a few thousand women from the strain of overwork. In the regulation by law of the hours which nurses may be called on to work the status of the whole profession is involved, and in this status the interests of the public are closely intertwined.

We have never admitted the claims of private-nurse-employers to escape from the limitations which it is proposed to place on authorities in institutions. Private nurses very generally take up their calling for life; institution nurses seldom for more than four or five years, unless they rise to the status of sisters, when their hours are by universal consent arranged on a fairly liberal basis. It is the private nurses who stand to lose their vitality, to find their general standard of health slowly deteriorating, and to experience a premature withdrawal from the enjoyment of service owing to overwork. Whether in long chronic cases, or in a succession of short acute ones, the single-handed nurse is required to carry an excessive load of fatigue and responsibility for an excessive number of hours without proper rest and with insufficient holidays. Unless this state of things can be dealt with legally, unless the Ministry of Health can make the life of the private nurse more tolerable, the nursing profession will never be restored to public favour as a desirable career. Out of the total number of candidates for training in nursing half at least will be found looking forward to the lucrative chances of private nursing. The women who have formerly made up this 50 per cent. of candidates are hearing too much altogether about the lives of private nurses, and are turning their ambitions in other directions.

To institute legislation for restricting nurses' hours is to lay hands on a very delicate piece of mechanism. It seems to us impossible to introduce a hard-and-fast rule of forty-eight hours a week to apply to every kind of nurse under every kind of condition. Certainly for private nurses and those in institutions the fortnight seems the better period of limitation. Is there not room for some discrimination of work? Eight hours spent in the operating-theatre, in the casualty department, in massage, is a widely different day's work to eight hours spent in varied duties of a domestic type. It may be necessary to arrange for longer spells of work, with a complete holiday afterwards, in some departments of nursing. There are puzzling matters to be thought out, which agree better with a department of health than with a department of labour.

The ideal arrangement is for the nursing service to be drawn closer and closer within the sphere of the Ministry of Health. The State nursing service, like the State medical service, is the dream of the future. But in the present we are confronted with a host of problems, none of which can be solved without the aid of skilled nurses. At every point in his plans the Health Minister is constrained to pause and reckon out what nursing aid he can depend on. Unless he is backed by a well-regulated, well-paid, and contented body of nurses, his activities in every single department will be perforce curtailed. The habit of overworking nurses is ingrained in the mind of the public notwithstanding all the indignation they are ready to manifest when facts are brought home to them. Nurses need protection by law from the very instincts of their vocation which prompt them to self-sacrifice for the sick. Without this safeguard the public will go on exploiting them, for none are so utterly stony-hearted as those who see their friends suffer and look to the nurse to bring relief; none are so forgetful of the claims of Nature as nurses can be in the hour of another's need.

